# Generating realistic artificial human genomes using adversarial autoencoders

**DOI:** 10.1093/nargab/lqaf101

**Published:** 2025-07-24

**Authors:** Callum Burnard, Alban Mancheron, William Ritchie

**Affiliations:** Institut de Génétique Humaine, 34094 Montpellier, France; Laboratoire d’Informatique, de Robotique et de Microélectronique de Montpellier, 34095 Montpellier, France; Laboratoire d’Informatique, de Robotique et de Microélectronique de Montpellier, 34095 Montpellier, France; Institut de Génétique Humaine, 34094 Montpellier, France

## Abstract

A publicly available human genome is both valuable to researchers and a risk for its donor. Many actors could exploit it to extract information about the donor’s health or that of their relatives. Recent efforts have employed artificial intelligence models to simulate genomic data, aiming to create synthetic datasets with scientific merit while preserving patient anonymity. Challenges arise due to the vast amount of data that constitute a complete human genome and the computational resources required. We present a dimension reduction method that combines artificial intelligence with our knowledge of *in vivo* mutation association mechanisms. This approach enables processing large amounts of data without significant computational resources. Our genome segmentation follows chromosomal recombination hotspots, closely resembling mutation transmission mechanisms. Data from the 1000 Genomes Project are used to train variational autoencoders with a Wasserstein GAN to generate novel data in a two-step process. After optimizing our strategy, our pipeline can generate a simulated population meeting several essential criteria. They are diverse but realistic; the newly generated combinations of mutations follow linkage disequilibrium found in humans. Our pipeline does not reveal the genetic identity of any individual donor, synthesizing genomes that differ from reference samples.

## Graphical Abstract

**Graphical Abstract:**
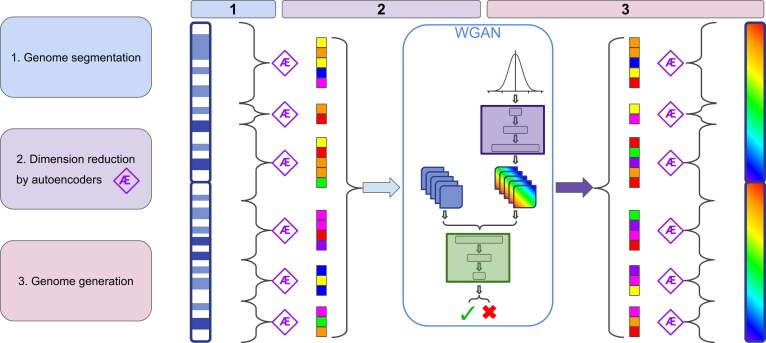

## Introduction

The sequence of nucleotides in the DNA contained in our cells constitutes the basis of the biological information necessary to characterize us as individuals, in combination with internal and external regulatory mechanisms. Over the past two decades, advances in sequencing technologies have allowed us to express these characteristics as variations from a reference genome, established by studying large swathes of the population [1].

We are all uniquely identified by these aspects, and it is becoming increasingly easy for healthcare services, research teams, and private companies to identify the genomic mutations underlying the phenotypes we observe. This leads to a trade-off, where these data can be used to treat diseases better, particularly when dealing with personalized medicine, but at the same time could have negative repercussions on the everyday lives of the bearers of these variations [2–4]. Great care is therefore needed when dealing with these data, in particular when sharing them with other actors.

The storage and sharing of medical data is highly regulated, especially in the European Union, where the authors are based [5]. However, it is more complex to protect these data when they are in use. Methods such as homomorphic encryption [6] or federated learning [7] could potentially alleviate privacy issues, but they are either very costly to implement or reduce the information that can be extracted from sequencing data.

A different approach to circumvent the privacy issue but still enable the use of genetic information for research purposes is to synthesize novel genomic data not directly derived from an individual’s sample. In the past, these methods have often focused on the simulation of an entire population and the coalescence of alleles within it [8–11]. In recent years, teams have sought to use available data describing the frequency of mutations in populations and create novel samples by assigning these mutations in novel combinations [12–14].

Artificial intelligence methods have been used to analyse genomic data to great success many times [15, 16]. Many of the most recent projects in genome generation use artificial intelligence methods, specifically generative approaches to determine appropriate combinations of mutations for sample generation [17], and incorporate more and more complex model architectures [18–21]. This approach produces diverse, realistic, and novel populations of genomes. In contrast to classical GWAS summary statistics that focus on individual variations, these artificial genomes capture the full genomic context such as haplotype structures, epistatic interactions, and non-linear relationships between variants. Synthetic genomes allow the creation of datasets enriched with specific rare variants or structural variations that may be underrepresented in real data. These can be used to assess their role in disease risk or therapeutic response and even allow testing of downstream hypotheses, such as variant effects on gene expression or alternative splicing. In addition, synthetic genomes can be tailored to underrepresented populations, addressing biases in current genomic datasets and enabling equitable research.

The main limitation, however, of contemporary AI methods is the size of both the training data and generated genomes. A classical artificial neural network is densely connected, meaning each neuron in a given layer is connected to each neuron of the following and previous layers. Increasing the number of dimensions in the sample used for training the model therefore leads to exponential increases in calculation cost.

We present here the haplotypic human genome generator (H2G2), a method to generate human genomic data on an increased scale by combating the curse of dimensionality. We first segment the data based on available biological knowledge, namely hotspots of recombination, and create a compressed representation of these subsections of data using dimension reduction methods. Then, these condensed genomic identities are processed by a generative neural network to simulate novel samples, while remaining coherent with the source dataset.

We use human genomic data from the 1000 Genomes Project [22], realigned onto HG38 [23], for its availability, high global diversity, and standardized methodologies. Mutation data for chromosome 1 were segmented based on hotspots of recombination likelihood as determined by a high-resolution map of recombination events established across thousands of detailed samples [24]. These recombination events dictate which mutations tend to be inherited together across generations, and therefore orchestrate the biological linkage between genomic variations at a large scale. Following this logic, mutations within a subsection share a higher correlation than mutations contained in two different subsections and allow for a more accurate compression of the data by dimension reduction methods.

We chose autoencoders to compress the data. These are a family of unsupervised machine learning methods using artificial neural networks to create latent representations of samples by compressing them more and more at each layer in the network [25, 26]. The models also implement a decoder to restore the compressed data to their full state, as faithful to the original samples as possible throughout the training process. Their strengths in dealing with any type of data, as well as handling non-linear datasets with variable multimodal distributions, are particularly relevant for this project.

The generation method we chose was the Wasserstein GANs [27, 28]. Their robust training process, by avoiding mode collapse and being less sensitive to hyperparameters compared to similar models, is particularly relevant for a project that seeks to increase the scale of data to be simulated. Using a large-scale generative model trained on encoded data (produced by discrete autoencoders) functions as a method of extending the generative neural network. From this point of view, each encoder serves as a local dense model that pre-processes data before feeding them to the generative model. Training them as separate autoencoders allows this step to be highly parallelized and can offer more insight into the performances of each local model.

We validated the realism of our simulated population through multiple measures such as mutation frequency, linkage disequilibrium, and statistics derived from population genetic tools. We also demonstrate that simulated genomes cannot be traced back to the specific training genomes from which they were generated.

## Materials and methods

### Python environment

Unless specified otherwise, all scripts were run in Python 3.8.10, using numpy 1.24.3, pandas 1.2.4, and keras 2.12.0 with tensorflow backend. Scripts run on the Genotoul cluster were launched using SLURM 22 and parallel release 20180122 [29].

### Code availability

The main scripts used during this project for data pre-processing, building ML models and the custom training loop for the WGAN (generative adversarial network using Wasserstein loss) model, are available at this GitHub repository: https://github.com/callum-b/H2G2/.

### Data

Mutation data for 2504 individuals in vcf format (presence/absence of mutations for each donor) as well as donor information come from the 1000 Genomes Project (phase 3) [22], realigned onto HG38 in [23]. They were downloaded from http://ftp.1000genomes.ebi.ac.uk/vol1/ftp/data_collections/1000_genomes_project/release/20190312_biallelic_SNV_and_INDEL/.

Each donor genome was separated into haplotypes. Mutations on chromosome 1 were filtered to only include those present in at least three of these haplotypes, so as to remove data with little detectable correlation with the rest of the dataset. Contiguous bins of a set number of mutations were then created from this list of variants.

Crossing over data were obtained from a high-definition map of recombination rates throughout the human genome. Ranges for chromosome 1 were extracted, and those with a recombination rate of ≤5 cM per Mb were removed to eliminate low-signal regions. The remaining ranges were reduced to their centres to create single base-pair loci that can be used as separators between sections of mutations, and these loci were sorted by recombination rate. Any locus within 5 kb of a higher intensity hotspot was removed, as this was considered to be diffuse signal around the hotspot rather than a separate event. This constitutes the preliminary filter step, before investigating the distribution of mutations between these hotspots.

Mutation data were then split into sections, bounded by hotspots, using these delimiters. Starting at one end of chromosome 1, mutations were added to a section until a hotspot was encountered, at which point the data collected thus far were saved to a file and a new section began. If a number of mutations that is lower than a user-set threshold was encountered, instead these mutations were appended to the previous section. If more than a maximum threshold of mutations were encountered, an ‘artificial hotspot’ was created, and data were saved to disk for this range. We used a hotspot-based segmentation method with minimum 500 mutations and maximum 5000 mutations per section. During this process, 43 artificial hotspots were thus created, and ∼2000 hotspots were ignored to not create sections containing low numbers of mutations. This choice of parameters was based on three factors: the number of subsections created, the size of these subsections (in number of mutations), and the distribution of accuracies of the autoencoders on them. In order to parallelize the training of the autoencoders, a large number of subsections is preferable. However, creating too many of these subsections leads to them containing few mutations each, which limits the compression factor that the autoencoders can apply to the data, and thus the power of this methodology. The parameters chosen here achieve a satisfactory balance between these two points, and produce high-accuracy models that can faithfully restore the mutation data.

### Autoencoders

Autoencoders were composed of an encoder with three hidden layers, then a bottleneck, and finally a mirrored encoder setup for the decoder. Each layer used ReLU (unless otherwise specified) for output, and there was a 20% dropout rate between the hidden layers in both encoder and decoder. Bottleneck size was defined as input size divided by 100, rounded up, and the size of the hidden layers was calculated using geometric spacing. We used reconstruction loss, measuring binary cross-entropy between the input data and the reconstruction data.

Variational autoencoders (VAEs) were composed of an encoder with three hidden layers, then a bottleneck, and finally a mirrored encoder setup for the decoder. Each layer was connected by sigmoid (for the final models used for compressing data), except for the bottleneck, which used linear activation, and the output layer, which used ReLU with a maximum value of 1. There was a 40% dropout rate in between each hidden layer for the encoder and the decoder. Bottleneck size was defined as input size divided by 100, rounded up, and the size of the hidden layers was calculated using geometric spacing. Its loss was measured as the sum of classical reconstruction loss and the Kullback–Leibler divergence [30] to ensure a locally continuous latent space.

Certain data types can call for the usage of convolutional neural networks [15, 16]. These are generally applicable for data that are variable in length, e.g. natural language processing. If this project were to be applied to full DNA sequences, they would be a very relevant inclusion. However, the mutation data used here can be expressed as fixed matrices of binary data, and therefore do not call for convolutional neural networks that add a lot of complexity to the model.

The training process for both these model architectures used a training set and a validation set, with an 80/20 split. The models were trained while monitoring loss on the validation dataset, and when this value increased (with a patience of 10 epochs, or 30 when using sigmoid and tanH activation due to slower convergence), training was halted and the weights of the model were restored to its best-performing iteration. The accuracy of this saved model on the validation dataset was used as its performance metric in the figures presented here.

### WGAN

The generative adversarial network used here is composed of two networks: a critic, which assigns a realness score to each sample it analyses, and a generator, which creates novel samples that attempt to fool the critic. It was implemented with the Wasserstein (earth mover) loss function, as well as a custom training loop so as to train the critic more than the generator, as per the usage of Wasserstein loss recommends. This loop also allows checkpoints to be saved during training.

The latent vector used contains 1000 random variables drawn from a Gaussian distribution with mean 0 and variance 1. The generator was composed of three hidden layers, using ReLU activation, their sizes determined by the total number of latent dimensions to predict, depending on the genomic sections processed. The first hidden layer is 10 times smaller, the second hidden layer is 5 times smaller, and the third hidden layer is 2 times smaller than that total. Finally, the output layer used the same size and activation function as the combined latent spaces of the autoencoders.

The critic was also composed of three hidden layers and an output, using ReLU activation, and their sizes also depended on the total number of latent dimensions considered. The first hidden layer was 10 times smaller, the second was 50 times smaller, and the third was 100 times smaller. The output layer is a single neuron with linear output.

The models, both the WGAN as a whole and the critic individually, were trained using RMSProp optimizer with a learning rate of 0.00005. In each training loop, the critic was trained five times more than the generator. We used Wasserstein distance, so the aim of the critic is to maximize the distance between the real samples and the simulated ones, and the aim of the generator is to minimize this distance. To implement this during training, the total loss of the critic was equal to its loss on simulated samples minus its loss on real samples, and the loss of the generator was equal to −1 times the loss calculated by the critic on the samples created by the generator for this training loop.

Multiple checkpoints of the model were sampled during training, and the data they produced were evaluated to find the optimal stopping point. Checkpoints were saved every 10 000 training epochs, and the results presented here were taken from the model trained for 30 000 epochs.

### Chromopainter

We estimated the ancestral diversity of our simulated genomes using Chromopainter [31], which attempts to assign sections of donor genomes to query genomes. We used this as a verisimilitude measure, as our simulated genomes should present approximately the same profile of crossing overs as the reference genomes.

We applied this tool to a reference query dataset, composed of three haplogenomes from each subpopulation listed in the 1000 Genomes Project phase 3 supplementary information, and to 100 randomly chosen simulated haplogenomes, for three genomic subsections. The donor dataset was composed of the 1000 Genomes Project individuals, without the members of the reference query dataset.

### Edit score

The edit score used here to compare simulated populations to reference populations was based on the Hamming distance metric [32]. We measured this distance for each simulated individual to each reference individual and retained the lowest of these values, which was then divided by the length of the sequence. This created an ‘edit rate’, which allowed comparison between sections of different sizes.

## Results

### Genome segmentation

The first step of our approach is to segment the genome into portions that are amenable to compression using autoencoders. To this end, we compared two segmentation approaches: a linear segmentation of the genome into equal-sized bins and a segmentation based on crossing-over hotspot regions. Recombination hotspots were used to segment mutation data for HG38 chromosome 1 (Supplementary Fig. S1A), setting minimum and maximum values for the number of mutations per subsection (Supplementary Fig. S1B). This method was compared to a naive method of simply separating mutations into bins of equal size. Autoencoders were then trained on the mutations contained in each subsection (Supplementary Fig. S1C) to compress the information contained in each one and reduce the dimensionality of the total dataset. Minor differences in reconstruction accuracy were observed on average between these segmentation methods, but further investigation was performed to identify the source of the drop in accuracy in the naive method.

**Figure 1.**
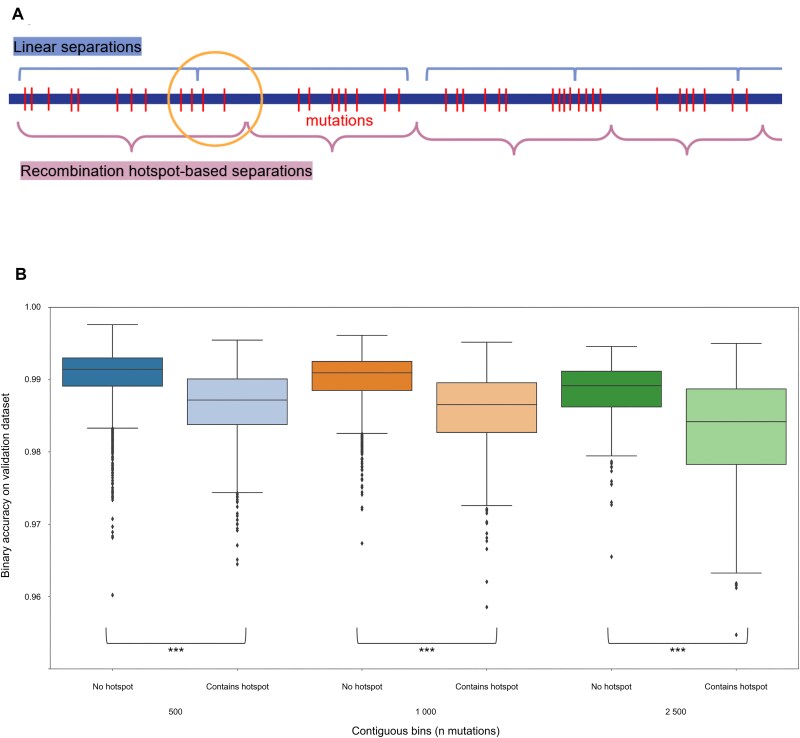
Genome segmentation based on recombination hotspots avoids drop in accuracy after autoencoding. (**A**) Linear splits of the genome may separate mutations that share more biological meaning with their neighbours on one side than on the other; recombination hotspot-based methods avoid this. (**B**) Boxplot showing the accuracy of autoencoder models that use linear separation. Bins where mutations were split by a hotspot (light colour) are less accurate than those that were not (dark colour). Statistical significance of the differences was tested using Mann–Whitney *U* test.

When using a naive approach (Fig. 1A), some mutations can become separated from others that share the most biological significance with them by chance. In contrast, the recombination hotspot-based method preserves these links. Analysing bins of mutations containing a strong hotspot (Fig. 1B) confirms this, as these sections that are split by a recombination hotspot show significantly lower reconstruction accuracy after autoencoding when compared to sections that do not contain a strong recombination hotspot. The hotspot-based segmentation methods reduce this source of errors by integrating fewer disruptive hotspots into the sections they produce (Supplementary Fig. S1D).

We used hotspot-based segregation of mutations for the rest of this study, allowing for a minimum of 500 and a maximum of 5000 mutations per subsection. This resulted in ∼2500 subsections across all of chromosome 1 (details in the ‘Materials and methods’ section).

### Dimension reduction by autoencoders

**Figure 2.**
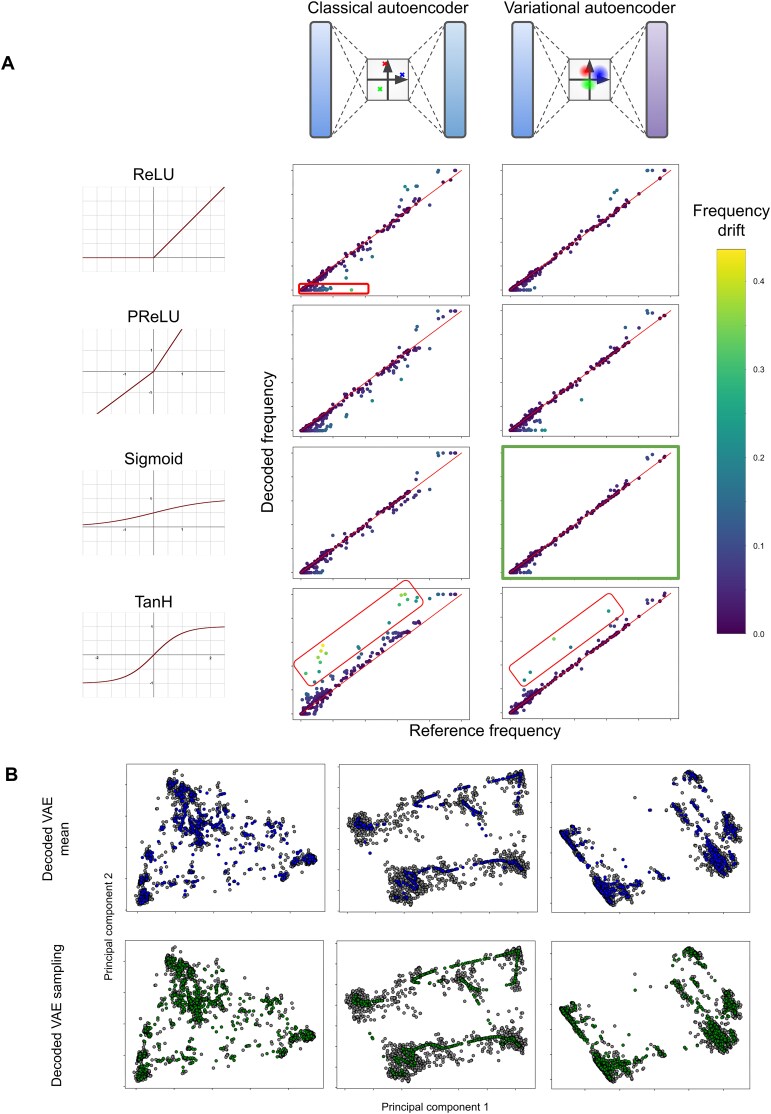
Dimension reduction by autoencoders. Model and hyperparameter optimization for accurate reconstruction. (**A**) Scatter plots comparing mutation frequencies in the reference dataset to the frequency of that mutation in the dataset after encoding and decoding by various autoencoder models. Occurrences where frequencies are equal align with red diagonal. Classical autoencoders and VAEs were tested, each with four different activation functions. For each plot, the frequency drift the absolute difference of the two frequencies. Particular drift patterns observed across different setups are highlighted manually. (**B**) Scatter plots of principal component analyses (PCA) of various datasets of genomic subsections. Each column is a different genomic subsection. In each plot, the reference dataset is shown as grey dots, and a dataset obtained after encoding then decoding by a VAE using sigmoid activation is shown in colour. Top row shows data obtained by decoding the mean of the projection of each individual in its latent space. Bottom row shows data obtained from the same VAE, but using a sampling around the mean of the projection of each individual in its latent space.

Segments generated from the previous section were then compressed using autoencoders (see the ‘Materials and methods’ section for full details). We first investigated the frequency of each mutation before and after compression via the autoencoders. We discovered that mutations with a low frequency in the reference dataset tend to be missing from the decoded dataset (Fig. 2A, upper left plot, highlighted by red box). This can be due to the trade-off between higher compression rates and encoding rare variations. The autoencoder is not learning the rare occurrences in which the mutation should be present as a reasonable trade-off for a gain in compression. The poor representation of rare mutations could also be due to neurons in the model ‘dying’, which can occur when using the ReLU activation function [33]. We therefore investigated multiple alternative activation functions, as well as a VAE [26]. Results in Fig. 2A show that different activation functions do indeed affect the number of disappearing mutations. Interestingly, the TanH activation function yields the fewest of these disappearing mutations, but also systematically tends to overestimate the frequency of mutations in the dataset (Fig. 2A, bottom row, highlighted in red).

Overall, using a VAE with either sigmoid or TanH activation function shows the lowest number of disappearing mutations as well as a low frequency drift (Supplementary Fig. S2A). Sigmoid activation function was chosen, so as to avoid the visible bias of highly overrepresented mutations that occur when using TanH, both in VAE and in classical AE.

PCA projections show that these VAE models are very good at reconstructing the population after dimension reduction (Fig. 2B and Supplementary Fig. S2B). Some do exhibit mode search, where the model simplifies the information it learns down to a set number of modes that once decoded, map to the main modes of the reference distribution.

### Genome generation

**Figure 3.**
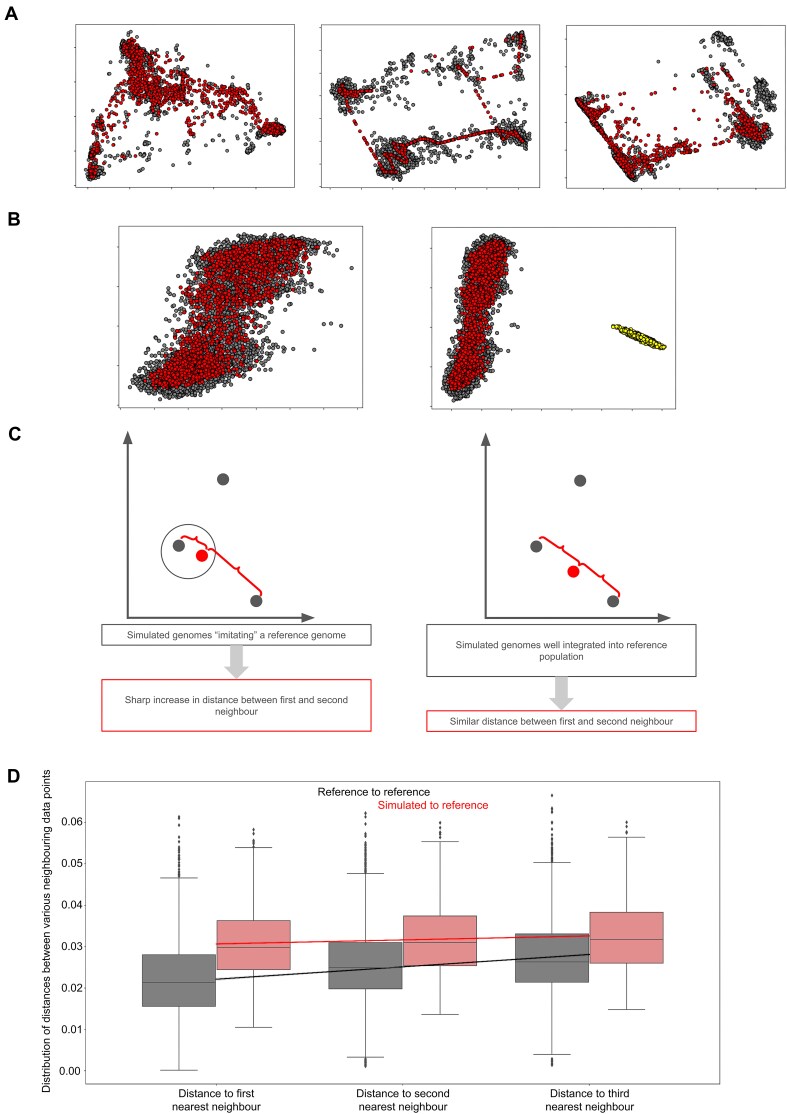
Statistical validation of simulated genomic segments. (**A**) Scatter plots of principal component analyses (PCA) of data generated by WGAN in red, versus reference data in grey for three genomic subsections. (**B**) Left: scatter plot of PCA of entire simulated genomic segment (red) versus entire reference genomic segment (grey). Right: identical PCA data to left panel, with the addition of shuffled simulated genomic segments (yellow). Each of the segments was generated independently and then assembled instead of being generated in one pass. This tests the coherence of the simulated dataset (red) that was generated in one pass. (**C**) Schematic representation of two possible scenarios for reidentification of a person’s genome. Left: the WGAN model produces synthetic genomic segments that are close replicas of samples it has seen in the training dataset and may thus compromise a patient's identity; in this case, there is a large difference between the simulated genome to its nearest neighbour when compared to its second nearest neighbour. Right: the WGAN model produces samples that are interpolations of multiple reference samples, meaning that distances between simulated genomic segments and their neighbours increase steadily. (**D**) Boxplot of the distribution of distances between reference genomic segments and their first, second, or third nearest neighbours (grey), and the same distances between synthetic genomic segments and their nearest neighbours in the reference dataset (red). Trend lines and average values are given for these distributions.

Following the segmentation and compression steps described above, we trained a WGAN on encoded subsections of genomic data spanning over 15 000 mutations in 13 subsections; this is equivalent to 1 Mb of DNA. After decoding these data and applying the same PCA projection, we can see that the samples generated by this model remain realistic at the subsection level. The coverage of the reference data is similar to that of the decoded data in Fig. 2B (Fig. 3A and Supplementary Fig. S3). Some regions exhibit a high level of mode searching (middle panel), which we attribute to low reference population diversity in this region, which limits the WGAN’s ability to create diverse novel samples for the region. To verify that the model can maintain this realism across multiple subsections, we performed a similar PCA projection (Fig. 3B, left, and Supplementary Fig. S4), this time comparing the full synthetic samples generated using this approach against the full reference samples. Additionally, we created a new dataset by shuffling the samples in each subsection and then concatenating these random assortments of genomic data. The shuffled subsections do not cluster with the reference and simulated data in this PCA (Fig. 3B, right) demonstrating that our synthetic population clearly conserves coherence across simulated genomic subsections more than expected by chance.

Although our simulated samples appear realistic, we need to verify that they are novel. To this end, we use the Hamming distance to measure the number of substitutions necessary to transform one sequence into another of the same length [32]. It was used here as a part of the edit score, which is the ratio between the Hamming distance from a simulated genome to its nearest neighbour in the reference dataset and the total length of the sequence. This score therefore ranges from 0 (identical sequences) to 1 (no common elements in sequence). When studying this score, it is important to pay attention to a possible phenomenon: if our generative model is ‘imitating’ a reference genome with minor edits, the edit score between the resulting simulated genome and its nearest neighbour will be very small, whereas the distance to its next nearest neighbour will show a sharp increase (see diagram in Fig. 3C, left). If our generative model is properly interpolating novel samples by combining information obtained from multiple reference genomes, the edit score between a simulated sample and its nearest neighbour and the edit score between the same simulated sample and its second nearest neighbour should not exhibit a sharp increase (Fig. 3C, right). These edit scores between different neighbours (Fig. 3D) demonstrate that our simulated genomes are novel samples (no edit scores of 0, therefore no identical copies of reference genomes). The trend lines for reference genomes and simulated ones correspond to the scenario (Fig. 3C, right) where simulated genomes are homogeneously integrated in the reference dataset, meaning that they are indeed created from multiple reference samples.

**Figure 4.**
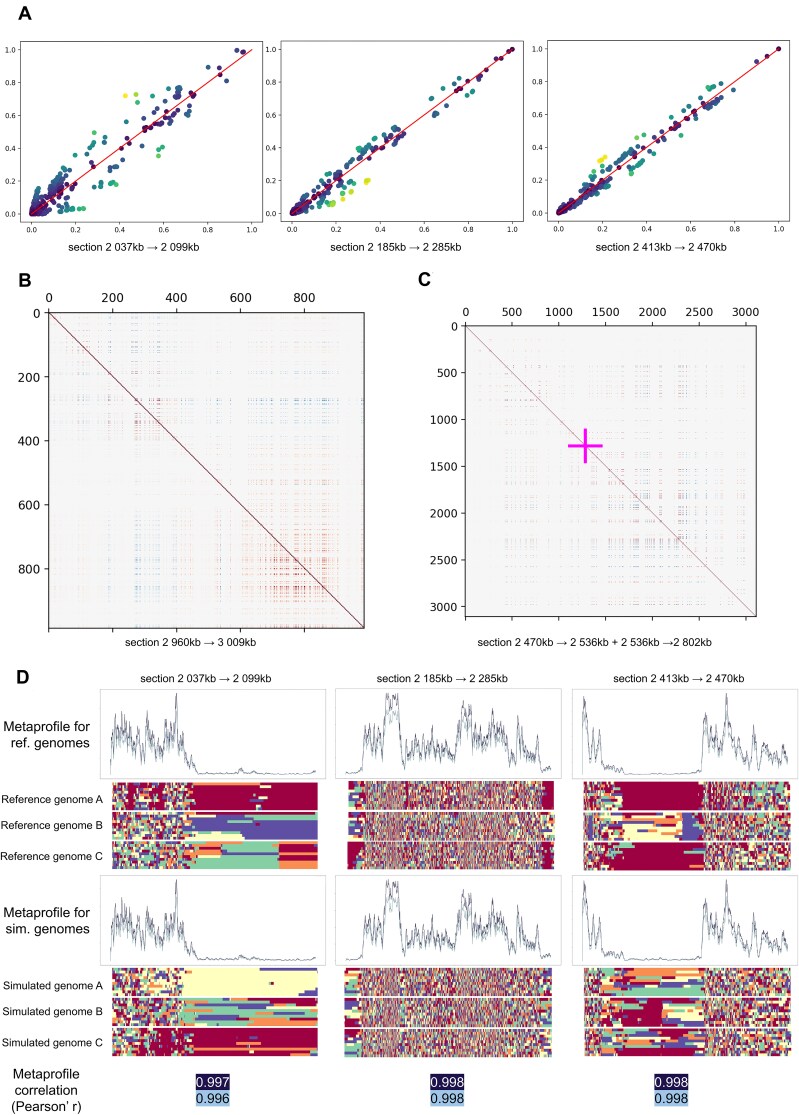
Biological validation of simulated genomic segments. Simulated data respect frequency, linkage disequilibrium (LD), and ancestry. (**A**) Scatter plots comparing mutation frequencies in the reference dataset to the frequency of that mutation in the dataset simulated using our pipeline. (**B**) Matrices showing LD between pairs of mutations in the reference datasets (lower triangle) and simulated datasets (upper triangle). Positive LD is represented in red and negative LD in blue. (**C**) LD matrices for pairs of mutations in reference and simulated datasets [same as for panel (B)] for two neighbouring genomic subsections. The position of the hotspot separating the two is indicated by the cross. (**D**) Chromopainter metaprofiles calculated over all samples for three genomic subsections, and three corresponding Chromopainter illustrated reconstructions from the datasets used to construct the metaprofiles. Chromopainter gives as output a series of reconstructions for each sample indicating the most probable ancestry for each mutation in the sample (red, Africa; orange, America; yellow, East Asia; green, Europe; blue, South Asia). Each plot is a different sample; each row in each plot is a reconstruction by Chromopainter. The horizontal axis is each mutation in the genomic subsection. Metaprofiles show how frequently this ancestry changes within samples. In dark blue, ancestry change from one individual to another; in light grey, ancestry change from one population to another.

The first step in exploring the biological validity of our simulated genomes is to compare the frequency of mutations appearing in this simulated dataset with their frequency in the reference dataset. This was done when measuring the performance of the various autoencoder models. However, here the evaluation is not as straightforward as for the autoencoder. Those models specifically should yield frequencies as close to those in the reference dataset, whereas a generative AI is designed to explore and create novel data. These mutation frequencies for the simulated dataset (Fig. 4A) show drift from the original frequencies, but they remain moderate. Mutations are reproduced at similar frequencies in the simulated population.

Next, we explore linkage disequilibrium (LD) between pairs of mutations in the reference dataset and our simulated one. Using a matrix (Fig. 4B) to compare this LD for mutations in a given subsection in the reference dataset (lower triangle) versus the LD in the synthetic dataset for the same pair of mutations (upper triangle). This shows that our models preserve these associations, both positive and negative. Expanding the same matrix to include two subsections shows a similar result for pairs of mutations in different subsections (Fig. 4C). LD here is, however, much weaker due to the presence of a recombination hotspot, which dampens the associations between mutations located on opposite sides of the hotspot (Supplementary Fig. S5A and B).

Finally, we used a population-based approach where we estimate how well the ancestry composition of the simulations matches that of the real genomes. To this end, we used a tool called Chromopainter that reconstitutes the genomic ancestry of a query population based on the mutation profile of a donor population. Chromopainter was applied to both 78 reference genomes (3 per sub-population listed in [23]) and 100 simulated genomes. From the raw results obtained, we established metaprofiles of ancestry switches, based on all reference or simulated genomes analysed (Fig. 4D). These metaprofiles show strong correlation between the reference and simulated datasets. Thus, our simulated population could realistically be obtained through population admixture *in vivo*.

## Discussion

In this study, we propose a novel approach for generating human genomic data at a large scale and to a high degree of fidelity by combating the curse of dimensionality. This pipeline combines a novel dimension reduction approach for data compression and a WGAN model to generate new samples. These allow a high level of parallelization and a reduction of computational resources required to generate genomes. Specifically, we show that our models can generate realistic and novel mutation data using a method that combines multiple neural network models in a novel manner, which can in theory be applied at any scale, and includes rarer mutations than previous AI-based methods [17, 18, 21]. Our technique is based on VAEs, which present many strengths as a dimension reduction method, such as representing non-linear data accurately within their encoding space and handling novel data points in a predictable manner. Additionally, we separate our data using recombination hotspots, which preserve the biological associations between mutations.

One of the main applications we envision for this sample generation pipeline is to create synthetic cohorts for specific sets of patients. A WGAN model trained to process general genomes could be specialized on a smaller dataset, obtained, for example, by a healthcare establishment sequencing all its patients bearing a disease or exhibiting similar traits. This generator would then create novel samples that follow the same implicit rules as the original patients in the cohort. This would allow sharing the cohort’s general information, which could be an important resource for research teams, without revealing the identity of any one person in it.

There are many other potential use cases for realistic synthetic genomes generated by AI. Creating a digital twin of an individual or a population could be useful for sharing or experimenting. Augmenting already available datasets of genomes might lend more robustness to certain research projects. Finally, the autoencoding of genomic data and its accurate reconstruction is a potential method of processing these samples securely, through federated learning or homomorphic encryption. The training of the autoencoders is separate from that of the generative model, therefore can be performed remotely by multiple teams (if coordinated properly) to increase the number of reference genomes included in the training data, leading to a federated learning approach for the final generative model. Additionally, the encoded training data used by the WGAN are meaningless without their corresponding autoencoders, and hence can be thought of as a form of encryption for sensitive patient data, if they are to be used to train a generative model like the one outlined here.

However, combining sequencing data obtained using different techniques will require caution as to the standardization methods used. Additionally, we have limited ourselves to short variations, whereas many large-scale, structural, and/or copy number variations also greatly impact the phenotype of the organisms bearing them.

The haplotypes used here as samples are based on available phasing data, which are not guaranteed to be completely accurate, especially at long range. It would be beneficial to projects like these to resolve long-range links between mutations, to present high-accuracy samples to our ML models. Haplotypes are also not consistent between chromosomes, as it is complex to establish biological linkage between mutations across chromosomes, but future applications may look to tie data from different chromosomes together. A project that can provide correlation data across chromosomes for certain populations would enable this scale to be explored.

The recombination map used to segment the genome here is based off of a study focused on an Icelandic population [24]. This represents a small subset of the human population, and therefore the hotspots identified are not accurate on a global scale. Not using them would still result in a loss of reconstruction accuracy for this methodology, as shown in the ‘Genome segmentation’ section of this project. Using them therefore constitutes a good starting point, but a similar study on a larger scale would be beneficial in establishing more widespread recombination hotspots.

VAEs using the sigmoid activation function were shown to be the best dimension reduction model among those we tested, but many more architectures could be tested in search of even greater efficiency and accuracy. As for generative models, these past years have shown remarkable progress in stable diffusion-based models that allow for detailed illustrations based on user specifications. Although these models are based on convolution neural networks, which are particularly well suited for processing images, which we do not use, it would be interesting to study their application to genomic datasets.

Evaluating the realism of a synthetic genome is a difficult task, in part because no standardized tools or methods exist for this purpose, and even more so for a synthetic population. The criteria proposed here—Chromopainter metaprofile correlation and edit score—all highlight various aspects of a simulated population that are desirable. To go further, it could be interesting to investigate the biological relevance of mutations contained in these simulated genomes and seek to measure degrees of similarity and difference using variations with known biological meaning.

## Supplementary Material

lqaf101_Supplemental_File

## Data Availability

The main scripts used during this project for data pre-processing, building ML models and the custom training loop for the WGAN model, are available at this github repository: https://github.com/callum-b/H2G2/ (also archived on Figshare: 10.6084/m9.figshare.29327531). Mutation data for 2504 individuals in vcf format (presence/absence of mutations for each donor) as well as donor information come from the 1000 Genomes Project (phase 3) [22], realigned onto HG38 in [23]. They were downloaded from http://ftp.1000genomes.ebi.ac.uk/vol1/ftp/data_collections/1000_genomes_project/release/20190312_biallelic_SNV_and_INDEL/.
