# Supplementary material for: Generating realistic artificial human genomes using adversarial autoencoders
Source: NAR Genom Bioinform. 2025 Jul 24;7(3):lqaf101. doi: 10.1093/nargab/lqaf101 (PMC12288873; doi:10.1093/nargab/lqaf101)
Supplement: lqaf101_Supplemental_File [file lqaf101_supplemental_file.pdf]

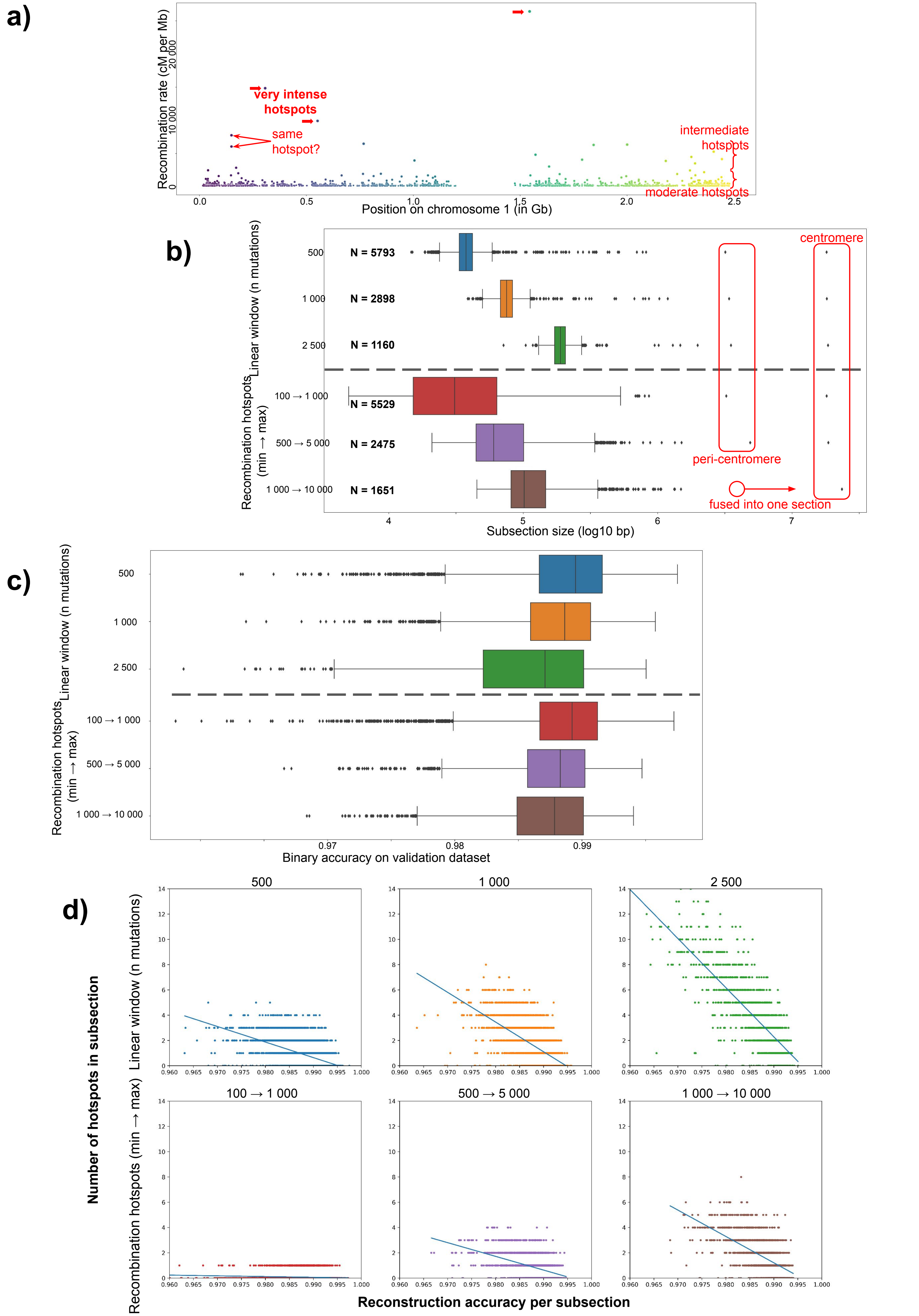
a)
Recombination rate (cM per Mb)
very intense hotspots
same hotspot?
intermediate hotspots
moderate hotspots
Position on chromosome 1 (in Gb)
b)
Linear window (n mutations)
Recombination hotspots (min → max)
N = 5793
N = 2898
N = 1160
N = 5529
N = 2475
N = 1651
centromere
peri-centromere
fused into one section
Subsection size (log10 bp)
c)
Binary accuracy on validation dataset
d)
Number of hotspots in subsection
Reconstruction accuracy per subsection

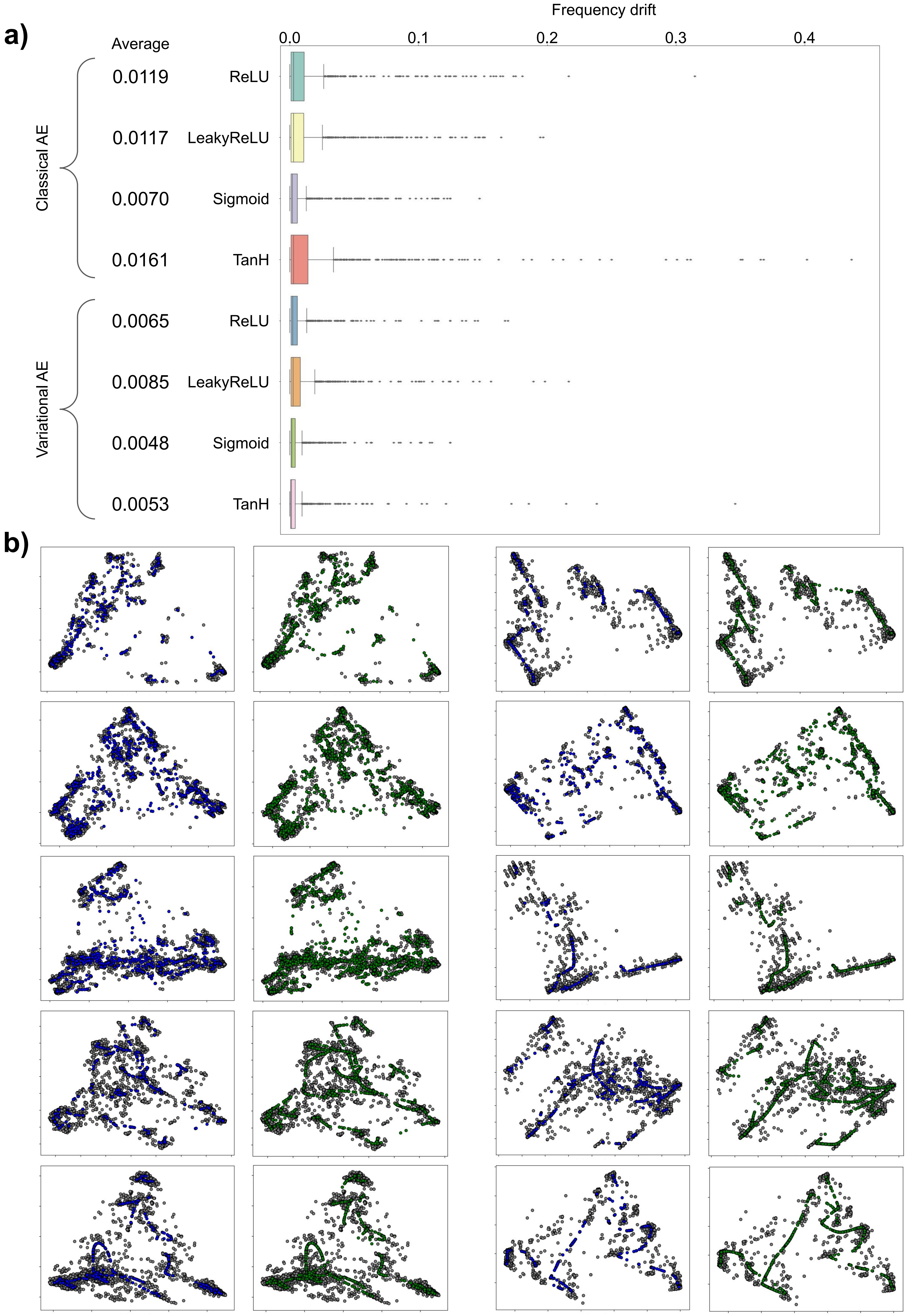

a)
Frequency drift
Average
0.0 0.1 0.2 0.3 0.4
Classical AE
0.0119 ReLU
0.0117 LeakyReLU
0.0070 Sigmoid
0.0161 TanH
Variational AE
0.0065 ReLU
0.0085 LeakyReLU
0.0048 Sigmoid
0.0053 TanH
b)

Decoded WGAN generation

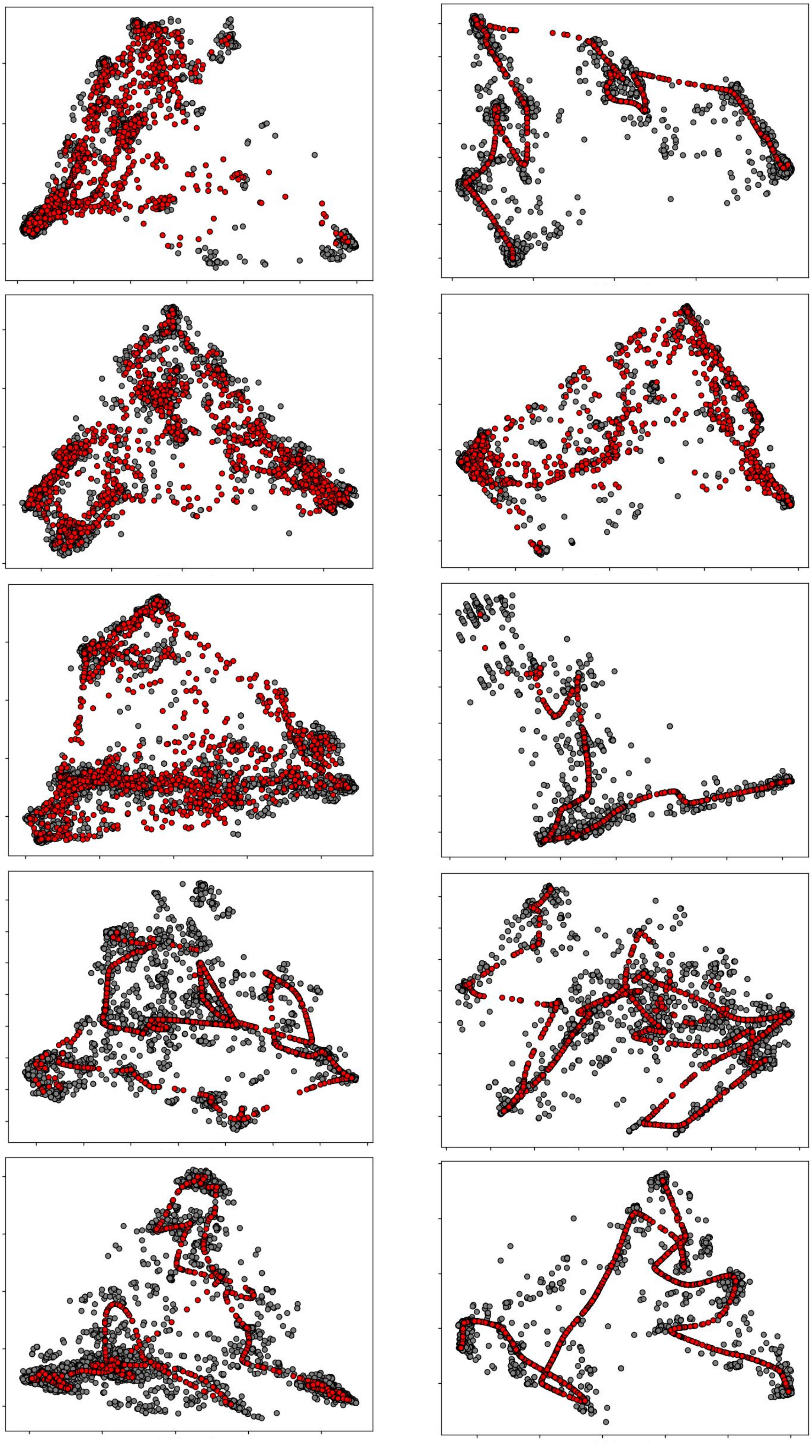

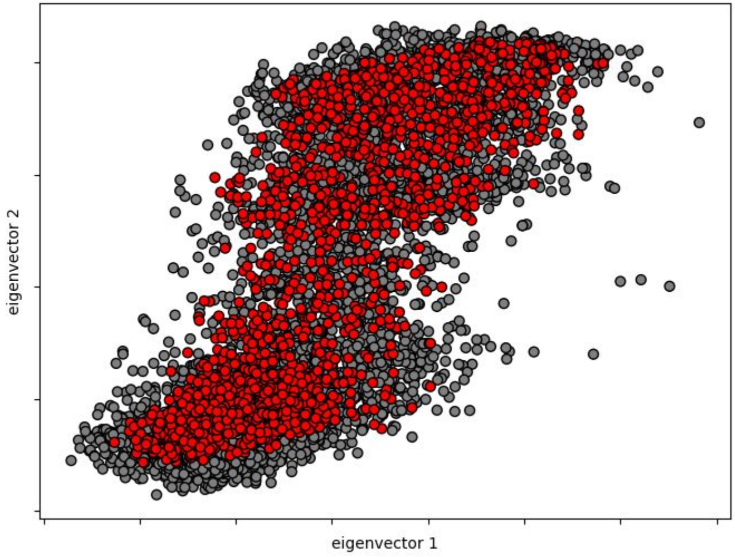
eigenvector 2
eigenvector 1

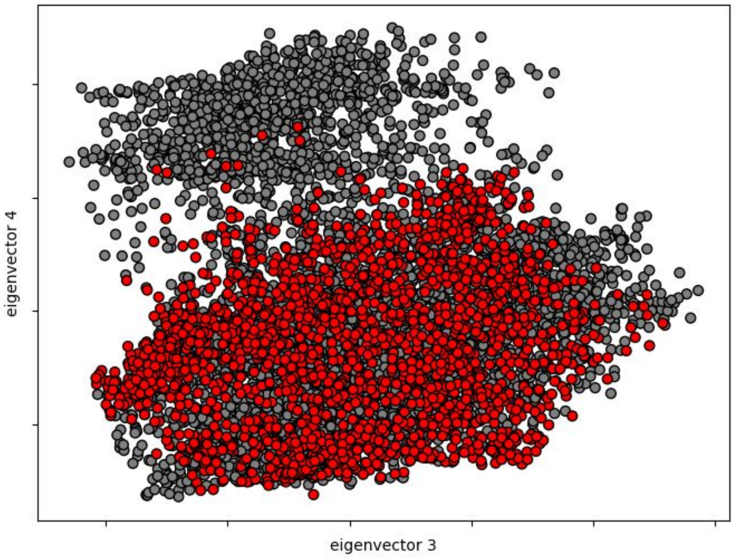
eigenvector 4
eigenvector 3

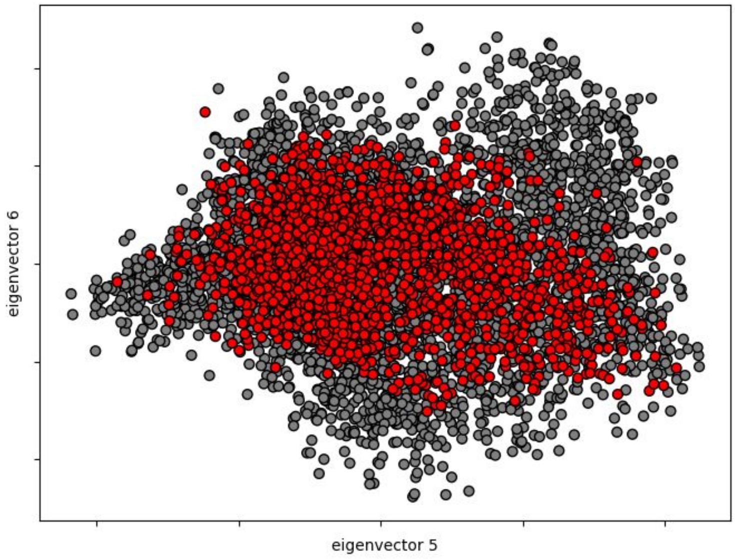
eigenvector 6
eigenvector 5

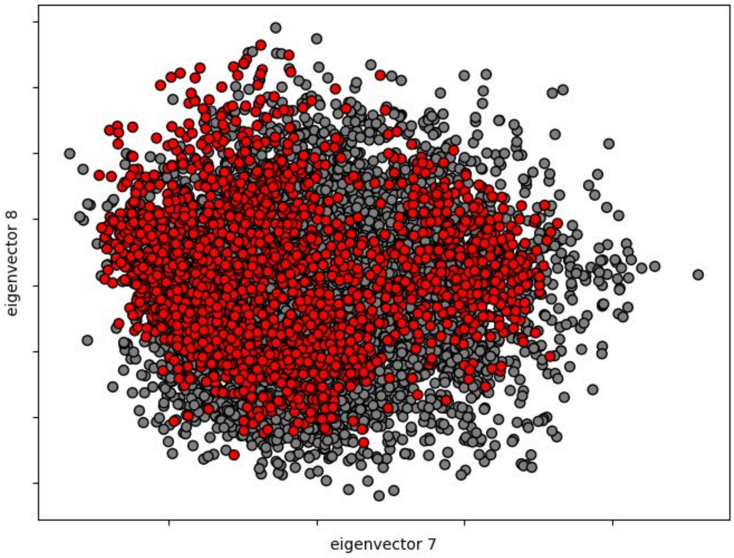
eigenvector 8
eigenvector 7

a)

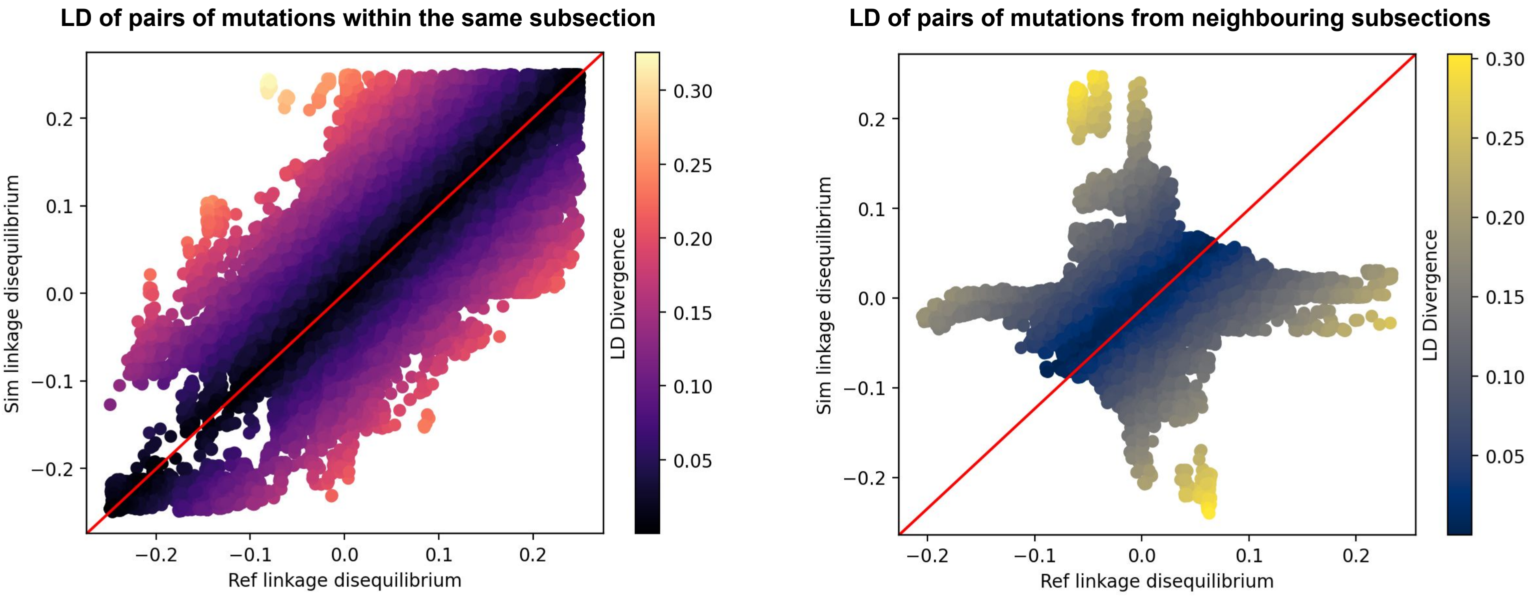
LD of pairs of mutations within the same subsection
Sim linkage disequilibrium
Ref linkage disequilibrium
LD Divergence
LD of pairs of mutations from neighbouring subsections
Sim linkage disequilibrium
Ref linkage disequilibrium
LD Divergence

b)

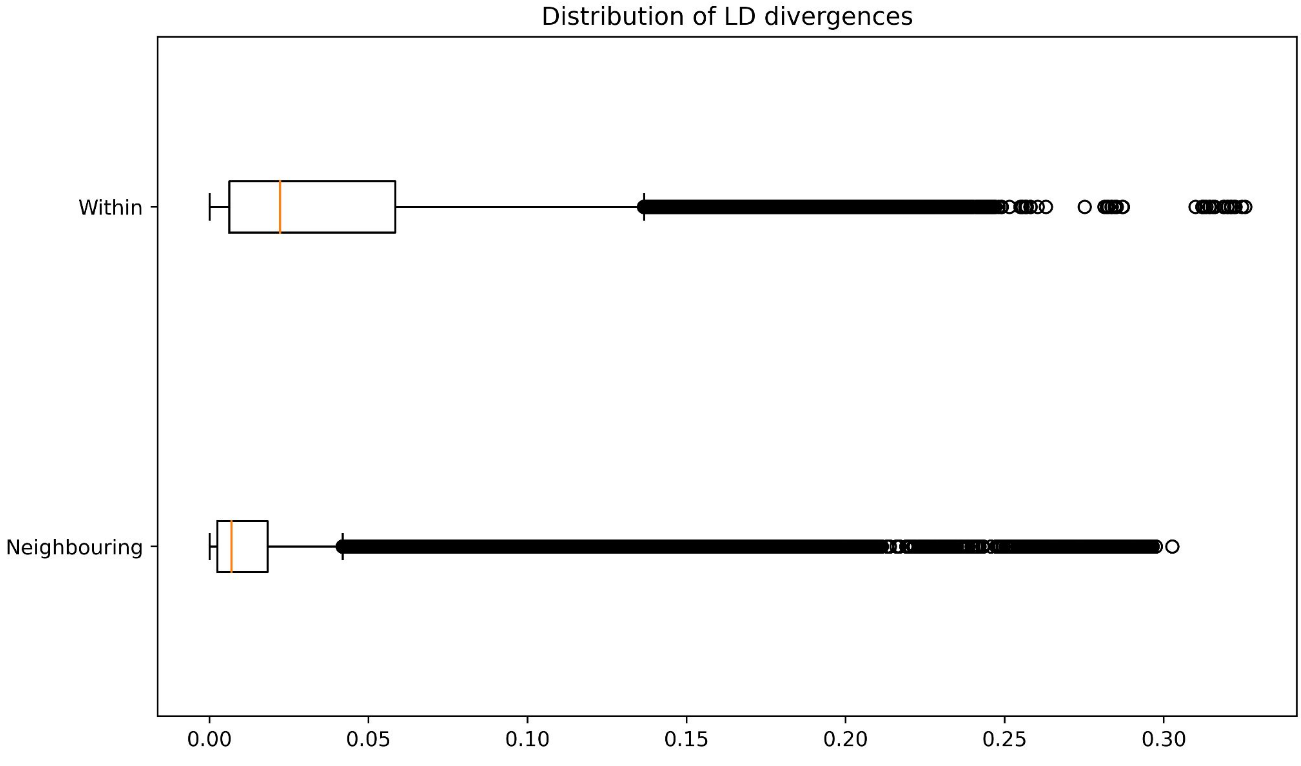
Distribution of LD divergences
Within
Neighbouring

Supplementary figure 1. (**A**) Scatter plot illustrating the distribution of the 2000 strongest recombination hotspots obtained after preliminary filtering along Human chromosome 1. Vertical axis is the recombination rate at that hotspot. Some individual hotspots or categories of hotspots are highlighted manually. (**B**) Boxplot of the size of genomic subsections depending on the segmentation method used. Interestingly, the centromere contains few listed mutations and therefore appears as one very large section. (**C**) Boxplot showing the accuracy of autoencoder models depending on the segmentation method used. From top to bottom: linear segmentation using 500, 1000 or 2500 mutations per bin, hotspot-based segmentation method allowing bins containing at minimum 100 and at maximum 1000, minimum 500 and maximum 5000, or minimum 1000 and maximum 10000 mutations per subsection. (**D**) Scatter plots comparing the reconstruction accuracy of an autoencoder applied to each subsection versus the number of hotspots in that section, for each genome segmentation method. Blue line is a polynomial fit to the data for each method.

Supplementary figure 2. (**A**) Boxplot of drift of each mutation for different autoencoder models. Average drift for each model is also included. (**B**) Scatter plots of Principal Component Analyses (PCA) of various datasets of genomic subsections. Each column is a different genomic subsection. In each plot, the reference dataset is shown as grey dots, and a dataset obtained after encoding then decoding by a Variational AutoEncoder (VAE) using Sigmoid activation is shown in colour. Blue dots show data obtained by decoding the mean of the projection of each individual in its latent space. Green dots show data obtained from the same VAE, but using a sampling around the mean of the projection of each individual in its latent space. All genomic subsections studied in detail that were not shown in fig 2b are shown here.

Supplementary figure 3. Scatter plots of Principal Component Analyses (PCA) of data generated by Generative Adversarial Network using Wasserstein loss (WGAN) in red, versus reference data in grey, showing principal components 1 to 8 for entire simulated genomic subsection. Variance explained by each PC: PC1: 0.2554567; PC2: 0.06295472; PC3: 0.03546104; PC4: 0.03267695; PC5: 0.02454073; PC6: 0.01836106; PC7: 0.01676653; PC8: 0.01407278.

Supplementary figure 4. Scatter plots of Principal Component Analyses (PCA) of data generated by Generative Adversarial Network using Wasserstein loss (WGAN) in red, versus

reference data in grey, with each row illustrating two different sections. All genomic subsections studied in detail that were not shown in fig 3a are shown here.

Supplementary figure 5. (**A**) Scatter plots comparing Linkage Disequilibrium (LD) values in the reference dataset and the simulated dataset. Comparison within the same subsection (left) and (right) in neighbouring subsections. For each plot, the colour of the dot indicates the absolute difference of the two LD values. (**B**) Boxplots showing the distribution of LD divergence (absolute difference of the reference LD value and the simulated LD value) for pairs of mutations found within the same genomic subsection (upper) or neighbouring genomic subsections (lower).
